# Burden of Disease and Unmet Needs in the Diagnosis and Management of Atopic Dermatitis in Diverse Skin Types in Australia

**DOI:** 10.3390/jcm12113812

**Published:** 2023-06-01

**Authors:** Ashling Courtney, Diego J. Lopez, Adrian J. Lowe, Zack Holmes, John C. Su

**Affiliations:** 1 Department of Dermatology, Eastern Health, Monash University, Melbourne, VIC 3128, Australia; 2 Murdoch Children’s Research Institute, Melbourne, VIC 3052, Australia; 3 Allergy and Lung Health Unit, University of Melbourne, Melbourne, VIC 3052, Australia; 4 Department of Dermatology, Alfred Health, Melbourne, VIC 3004, Australia

**Keywords:** atopic dermatitis, eczema, skin of colour, ethnic skin, healthcare disparity, Aboriginal and Torres Strait Islander peoples, Indigenous Australian, First Nations

## Abstract

Atopic dermatitis (AD) is a common, chronic, inflammatory skin disease affecting Australians of all ages, races, ethnicities, and social classes. Significant physical, psychosocial, and financial burdens to both individuals and Australian communities have been demonstrated. This narrative review highlights knowledge gaps for AD in Australian skin of colour. We searched PubMed, Wiley Online Library, and Cochrane Library databases for review articles, systematic reviews, and cross-sectional and observational studies relating to AD in Australia for skin of colour and for different ethnicities. Statistical data from the Australian Institute of Health and Welfare and the Australian Bureau of Statistics was collected. In recent years, there has been substantially increased awareness of and research into skin infections, such as scabies and impetigo, among various Australian subpopulations. Many such infections disproportionately affect First Nations Peoples. However, data for AD itself in these groups are limited. There is also little written regarding AD in recent, racially diverse immigrants with skin of colour. Areas for future research include AD epidemiology and AD phenotypes for First Nations Peoples and AD trajectories for non-Caucasian immigrants. We also note the evident disparity in both the level of understanding and the management standards of AD between urban and remote communities in Australia. This discrepancy relates to a relative lack of healthcare resources in marginalised communities. First Nations Peoples in particular experience socioeconomic disadvantage, have worse health outcomes, and experience healthcare inequality in Australia. Barriers to effective AD management must be identified and responsibly addressed for socioeconomically disadvantaged and remote-living communities to achieve healthcare equity.

## 1. Introduction

Atopic dermatitis (AD) is a common, chronic, relapsing inflammatory skin disease that imposes a high burden on Australian families and on healthcare [1,2,3]. People of all ages, races, ethnic groups, and skin colours are affected [3,4]. In recent decades, the prevalence of AD and atopic disease has risen in Western societies [5].

Australia is a multicultural country with a population of over 25 million, and it is estimated that over 6 million of those individuals have skin of colour [6]. Skin of colour refers to skin with increased melanin and darker pigmentation. Typically people from ethnic and racial groups such as Asian, African, Latinx, Middle Eastern, and First Nations Peoples have skin of colour [7]. Throughout history, Australia has experienced a net gain in population due to waves of migration, particularly related to Western settlement, the world wars, and various international economic and humanitarian events. Respective immigrants have substantially outnumbered emigrants and contributed to the increasing diversity of Australia’s population [6,8]. The proportion of Australia’s population born overseas was 30% in 2020, with England (3.8%), India (2.8%), China (2.5%), New Zealand (2.2%), and the Philippines (1.1%) being the most represented countries [8]. The Australian 2021 Census found that 812,728 people identified as Aboriginal and/or Torres Strait Islander (First Nations Peoples) [9]. This is an increase of >25% over a five-year period, with First Nations Peoples now representing >3% of the Australian population [9]. There is limited information about AD prevalence and incidence in the literature on non-Caucasian people in Australia.

Particular challenges underscore the management of AD in First Nations Peoples and Australian ethnic minority groups. First, little is known about the burden of disease of AD in these groups in Australia [10]. Second, although phenotypic and biomarker variations between racial and ethnic groups are known to potentially affect diagnostic accuracy and can guide the choice of therapy, these are not well understood in these groups [4,11]. Third, the standard model of care in managing AD is often culturally inappropriate or inaccessible for socioeconomically disadvantaged and diverse multi-ethnic communities [2,12]. Reduced access to local healthcare and the unaffordability of treatment options are key barriers to effective treatment for these groups. Fourth, while there is a need for greater inclusion of skin of colour in clinical trials for new therapeutic agents, reduced access to trials for social, cultural, linguistic, or geographic reasons may result in the ongoing under-representation of certain minority groups [11,13]. The resulting impact of living with a poorly managed chronic skin condition may, in turn, exacerbate issues with self-esteem, social isolation, stigmatisation, and discrimination, especially within communities already relatively lacking understanding, education, and awareness of AD co-morbidities and best practices [2,14].

## 2. Burden of Disease in Aboriginal and Torres Strait Islander Peoples in Australia

Globally, AD affects a substantial proportion of children, with considerable variability across countries [15]. The severity of AD also varies across age groups and geographic regions [15]. These variations may result from reported differences in disease onset, persistence, presentation, and timely diagnosis due to the phenotypic heterogeneity of AD across ethnicities and races [4,11,15]. Some of this variability may also be explained by cultural differences in survey responses or disparities in health care resulting from impaired access or suboptimal diagnosis and management [15].

Both the prevalence and relevant disease burdens associated with Australian subpopulations have changed in recent years. The Australian Institute of Health and Welfare reports that between 2003 and 2018, there was a 44% increase in the total number of disability-adjusted life years (DALY) among First Nations Peoples [10]. The main factors contributing to this worsening in disease burden were population growth (the First Nations population increased by 40% between 2003 and 2018), ageing, and changes in the amount of disease and injury [10]. This applied to almost all conditions examined in this subpopulation with the exception of infectious diseases, which showed a decline in DALY, mainly due to a reduction in the amount of disease [10]. Notably, there was no change in the amount of disease over time reported for dermatitis and eczema, some mental illnesses, and substance use disorders, notwithstanding potential ongoing under-reporting of these conditions and data gaps for AD prevalence in First Nations communities [10].

There have been conflicting data published on the burden of other atopic diseases on Australian First Nations Peoples. A study from over 20 years ago reported that the prevalence of asthma in rural First Nations adults was low when compared with non-Indigenous Australians; asthma prevalence in First Nations children was described as negligible [16]. A more recent study, however, reported that asthma affects over 15% of Australian First Nations people, with at least 50% higher prevalence than that for non-Indigenous Australians [17]. It is unclear how much of this apparent change in observed asthma prevalence can be attributed to evolving environmental and socioeconomic factors that influence the acquisition of atopy or, in fact, to improved disease recognition and reporting. However, despite the fact that Australia is one of the countries most affected by the food allergy epidemic, there are no reports on food allergy prevalence for First Nations Australians [18]. Data on the apparently changing prevalence of other atopic diseases, however, does not necessarily imply corresponding changes in AD prevalence.

An article published in 2020 focusing on the Northern Territory (NT), describes health disparities among many of Australia’s remote-dwelling First Nations Peoples [19]. The article suggested that First Nations Australians are more likely to be affected by disease in several vital organ systems at rates exceeding those seen in their non-Indigenous counterparts [19]. Regarding Aboriginal cutaneous diseases in the remote NT, this article only reports infections, in particular, disproportionate rates of scabies and impetigo, endemic conditions increasingly studied in recent years [19,20,21]. However, as for much of the general literature, there is no mention of atopic disease, eczema, or dermatitis [19,20,21].

Data for the burden of AD in remote living First Nations Peoples are limited (Table 1). A study looking at dermatological disease requiring specialist care in the Kimberley region of Western Australia over a five-year period found that among Aboriginal patients, the most common conditions were eczema and dermatitis (19%) [22]. A study conducted in Australia of First Nations dermatologic presentations to an urban specialist clinic within an Aboriginal Health Service found that eczema and benign, premalignant, or malignant neoplasms were the most common [23]. This study was the largest of its kind at the time of publication in 2021 [23]. There is a growing body of evidence that there is a significant burden of AD and bacterial skin infection among urban-living First Nations children and young people in high-income countries including Australia [24]. It also appears that First Nations Australians may experience more severe symptoms than their non-Indigenous peers [24]. Less is known about AD in remote-living First Nations Australians, but it is likely that its burden of disease is presently under-estimated and it may have worse outcomes compared with urban disease, due to reduced access to healthcare [19]. A search was conducted in Pubmed, Wiley Online, and Cochrane Library databases between 2000 and 2023 using the keywords ‘atopic dermatitis’, ‘eczema’, ‘ethnic’, ‘First Nations’, ‘Indigenous’, ‘Aboriginal’ and ‘Australia’ yielded 92 results in total. Only 19 of these were found to be relevant to this topic; however, none specifically discuss the burden of AD in rural First Nations communities in Australia. Therefore, it is an area that warrants urgent attention.

## 3. Burden of Disease in Other Ethnic Groups in Australia

Although most AD studies have focussed on Caucasian populations, AD is in fact more prevalent in Asian and Black skin [4]. Australian-born children of Asian-born mothers in their first six years of life have a higher burden than Caucasian children for most allergic diseases including AD and nut allergy [32,33,34]. Melbourne researchers, however, found a lower risk of food allergy and eczema in Asian-born children, who subsequently migrated to Australia at the age of five years, compared with Australian-born children, whether of Asian or non-Asian ethnicity [33,34,35]. Another Melbourne-based study found that high peanut allergy prevalence among infants of Asian-born parents appears to have occurred in a single generation and was not present among infants with parents migrating from other countries [34]. This increased prevalence of allergic disease in children of Asian ancestry, but born in Australia, is not completely understood. Suggested factors contributing to this difference observed between the cohorts include the ‘hygiene hypothesis’ and differences in humidity, ultraviolet light, and vitamin D exposure in early life [33]. Both genetic and environmental factors therefore likely alter the development of AD, nut allergy, and other atopic diseases in Australia [33,34,35,36].

In appraising disease burden data, one is mindful that cultural elements can affect both research engagement and health-seeking behaviour. The latter in turn shapes real-world data sets. As previously mentioned, in 2020 30% of the Australian population were born overseas [8]. One-in-five first and second-generation Australians speak English as their second language at home [12]. These culturally and linguistically diverse Australians face barriers to accessing both health services and research studies [12]. Barriers include poor health literacy, language and communication difficulties, lifestyle differences, and financial constraints on the prioritisation of and access to healthcare [12]. People from culturally diverse backgrounds face multiple challenges in accessing multilevel health services such as those required for the care of moderate-to-severe atopic disease. The prevalence of AD may therefore be significantly under-estimated for these groups.

## 4. AD Characteristics in Aboriginal and Torres Strait Islander Skin

AD is a heterogeneous disease with significant differences in prevalence, genetic background, and immune activation patterns, depending on the racial group [11,37]. Overall rates of AD in Africa and Oceania are higher compared with India and Northern and Eastern Europe [38]. Studies have consistently found filaggrin loss-of-function mutations in around 50% of Europeans and 27% of Asians with AD [11]. These mutations were six times less common in African American than in European American patients, even in patients with severe disease [11]. Known filaggrin mutations, therefore, seem to play less of a pathogenic role in AD for Africans compared with Europeans and Asians [11]. However, uncommonly recognised filaggrin variations have been found in AD of African Americans and greater use of advanced sequencing may be required to determine the role of filaggrin in non-Caucasian AD [39]. Strong T helper 2 cells (T_H_2) activation is apparent in AD for all ethnic groups, but important differences in immune polarisation exist between the different ethnicities, including evidence of more T helper 17 cells (T_H_17) activation in skin of colour [11]. Filaggrin genotypes and immunophenotypes for AD in First Nations Australians have not yet been characterised.

The Melbourne Atopy Cohort Study (MACS) has sought to characterise AD risk factors, clinical presentations, natural history, allergic comorbidities (eczema, food allergy, asthma, and allergic rhinitis), and their outcomes in Australia [40,41]. As a longitudinal single-centre study based in Melbourne of a high-risk birth cohort and their families, the MACS study has recently identified five distinct eczema subclasses with associated risk factors and allergic outcomes not dissimilar to those identified in Europe, the United Kingdom, and the United States [36,40,41]. The specific AD trajectories of First Nations Peoples have not been characterised yet. Further studies are underway to clarify AD trajectories for First Nations and rural Australian communities.

Scabies and pyogenic infections present challenges for AD diagnosis and the determination of AD prevalence in First Nations communities. This is due to their phenotypic overlap with AD and augments the diagnostic difficulties already presented by AD in skin of colour. Erythema classically associated with AD may be misappropriated as erythema associated with infection; alternatively, erythema may be absent or appear violaceous in skin of colour thus leading to underdiagnosis of AD (see below). Compared with scabies in the general Australian community, Norwegian scabies, and more severe scabetic phenotypes are much more common in First Nations Peoples and may mask an underlying diagnosis of AD [20]. Likewise, pyogenic infections are common to the point of normalisation in some Aboriginal communities [21]. It is unknown how much this difference relates to genetic or biological factors, younger demographics, climatic distinctions, or other social factors like overcrowded housing and poor treatment access [12,15]. As the age of onset, severity, and persistence of both AD and concurrent infections are likely influenced by complex genetic and environmental interactions, stratification for all relevant variables will be critical in future studies to identify strategies for the better diagnosis and treatment of both problems.

## 5. Differences in Clinical Phenotype of AD in Australian Skin

Knowledge and awareness of the differences in presentation and clinical phenotypes of AD within the various ethnic groups in Australia are important for accurate early diagnosis and appropriate management. There are clinically significant subtleties in the visual appearance of eczema which are predominantly due to differences in pigmentation, lesional morphologies, and lesional distribution [4,42].

The classical features of AD are well described in the literature and typically relate to Caucasian white skin [4,11,42]. Despite a predominance of studies in individuals with white skin, AD has been found to occur with increased frequency, severity, and persistence in skin of colour [11,42,43]. When describing AD in skin of colour in the literature, most studies refer to Black and Asian individuals [11,42]. The skin of Australian First Nations People varies greatly between regions due to variations in parental lineage and parents of mixed races are common. Darker skin pigmentation, Fitzpatrick type 5 and 6, is more commonly seen in certain regions including the Northern Territory, North Queensland, and the Pilbara region of Western Australia [44].

A study characterising the Asian AD skin phenotype, describes increased hyperplasia, parakeratosis, higher T_H_17 activation, and a strong T_H_2 component [45]. This correlated with a phenotype combining features seen in European American patients with AD and those with psoriasis [45]. In clinical practice, Asian AD commonly presents with more well-demarcated lesions, increased scaling, and lichenification when compared with typical Caucasian AD [4].

In individuals with darker skin pigmentation, AD presents more often with extensor and perifollicular involvement, and sometimes primarily with papules [42]. Classical bright red skin erythema, which is often seen to be a hallmark of atopic dermatitis in individuals with lighter skin tones, is more likely to appear violaceous or it may be difficult to appreciate at all in darker skin [42,46]. As a consequence, the use of standardised scoring systems that rely on skin erythema, including SCORing Atopic Dermatitis and Eczema Area and Severity Index, may result in less experienced physicians significantly underappreciating the severity of AD in darker skin [4,39.40]. Other classic findings that are more commonly reported in skin of colour include Dennie-Morgan lines, diffuse xerosis, and hyperlinearity of the palms [11,47].

Patients with darker skin are also more prone to prurigo nodularis compared to patients with white skin [11,47]. This type of presentation may relate to increased pruritus, greater inflammation, and increased rubbing and scratching [47,48]. Post-inflammatory hypo- and hyper-pigmentation occur more frequently in darker skin types and are more pronounced than in white skin [49]. Changes in skin pigmentation may return to normal within weeks to months with the exception of chronic excoriated skin secondary to severe eczema, which may result in permanent depigmentation [4]. The resulting dyspigmentation can result in psychological distress, poor-self image, social stigmatisation, and psychiatric comorbidities [14].

Despite the high prevalence of AD in skin of colour, there is little published literature describing the clinical features of AD in ethnic groups such as Latinx, Middle Eastern, and First Nations Peoples [43,50,51]. Specifically, detailed descriptions of the various clinical presentations of AD in different age groups in Australian First Nations Peoples, unfortunately, remain absent. Beyond differences in clinical appearance, AD may be physiologically and immunologically distinct in these ethnic groups, and respective responses to standard therapies are also largely unstudied. There is a similar paucity of information regarding genetic risk factors and polymorphisms that influence susceptibility to AD in Latinx, Middle Eastern, and First Nations populations [43]. Distinctions in skin biology, immune phenotypes, and ideal targets for therapy in these subpopulations are incompletely understood. By contrast, there is an abundance of evidence that adverse socioeconomic, environmental, and healthcare factors influence AD prevalence, severity, and persistence, and these same risk factors are more common among ethnic minority populations [43]. A recent article aiming to reframe understandings of factors underlying racial and ethnic disparities in AD care examining Black and Latinx populations contrasts the tendency for researchers to pursue biological (genetic, clinical, or immunophenotypic) explanations for observed health outcome disparities with evidence that conversely points to contextual differences (socioeconomic status, racism, environmental exposures, and healthcare gaps) as the real primary reasons for such disparities. [43]. The same framework could potentially be applied to First Nations Peoples in Australia for whom many social disadvantages remain. Future research is warranted to not only explore clinically significant differences in AD phenotypes in skin of colour, but also the effects of contextual factors both on AD phenotype and ultimately, on AD health outcomes, to help direct personal and public health interventions in Australia’s growing and uniquely diverse population.

## 6. Unmet Needs in the Management of AD in Diverse Skin Colours in Australia

As such, AD is a highly heterogeneous disease and requires a multifaceted management approach both at the personal and community levels [52]. Standard approaches to AD management may not be the appropriate model of care for minority ethnic skin types [4]. For skin of colour, not only is AD more prevalent and its propensity to develop and its treatment response influenced by genetic factors, but cultural and socioeconomic factors play vital roles in its evolution and outcomes [47].

A recent narrative review details the management of AD in Australia, from general skin care measures to more complex targeted therapies [52]. Certain subpopulations, including First Nations Peoples, face greater challenges in avoiding exacerbating environmental factors, such as extremes of temperature and humidity, irritants and allergens, alkaline detergents, and abrasive clothing due to social and geographic factors such as rurality and limited access to appropriate healthcare resources [52]. Additional factors contributing to socioeconomic disadvantage disproportionately affecting First Nations Australians and creating barriers to effective AD care include the costs of transport, primary and specialist dermatological care, prescription treatments, and expensive skin care regimens [23,24,53,54]. Although healthcare costs are higher in First Nations Australians, disposable income available for healthcare is lower [53]. Notwithstanding current government incentives in place, the financial burden for individuals and families in accessing treatment remains an important obstacle to address in bridging the gaps in Australian healthcare.

Access to specialist dermatological services remains limited for rural communities where many First Nations Australians and recent immigrants live. There is a rapidly evolving landscape of new AD therapies requiring specialist input for access [13,52]. The majority of patients with AD can be effectively managed with education and good adherence to trigger avoidance, emollients, topical anti-inflammatory agents, and phototherapy [52]. However, some moderate to severe or treatment-resistant AD requires the consideration of systemic immunosuppressive agents [13,52]. Both phototherapy and systemic therapies (including newer biologic and small molecule therapies) generally require specialist dermatologist supervision. In addition, skin care education at the primary care level is hampered by primary care time and access constraints due to a critical shortage of rural general practitioners. Although there are some visiting dermatological services in place and some telehealth services are available, most rural communities still face major barriers to timely specialist care [22].

Acknowledgement of common cultural practices and traditional herbal remedies used for the treatment of AD is also important [4]. In Asian, African, and Hispanic cultures, patients often use homeopathic remedies for skin problems prior to and in conjunction with seeking specialist care, which may have significant implications on treatment outcomes [4]. Delaying treatment with homeopathic remedies may also serve to worsen AD by allowing for the development of potential skin infections, scarring, and chronic hypo- or hyperpigmentation. While some cultural remedies have been shown to be beneficial in AD, the associated risks are largely unknown, and therefore education about the benefits of early evidence-based treatment and prescription medication is important [4].

An increasing body of literature describes underreporting of race and ethnicity, and the overrepresentation of White individuals in clinical trials [55,56]. There is little data on the efficacy of common therapies for AD in skin of colour [4]. This is partly due to the underrepresentation of some racial and ethnic groups in clinical trials [13,55,56].

A study of the representation of racial and ethnic minorities in clinical trials for AD published between 2000 and 2009, found only 59.5% of studies included race and ethnicity as baseline demographic information [57]. Of the studies reporting race or ethnicity, the subject population included 62.1% white, 18.0% black, 6.9% Asian, and 2.0% Hispanic [57]. Only 10.3% of studies comment on race or ethnicity in the interpretation of results, making it difficult to apply the results to other minority ethnic groups [57]. As all Australian dermatology trial centres are in urban areas, rural subpopulations including many First Nations communities have minimal access to these. Future research into the treatment of AD should clarify the role of particular molecular targets in skin of colour [4,13]. This is crucial to personalising treatment options for AD [55,56]. For this to happen, there needs to be greater inclusion of multi-ethnic skin types in clinical trials.

## 7. Impact of Unmet Needs in the Management of AD

Undertreated AD can have profound debilitating life burden on those affected, their carers, and their families [1,2,14,58]. “Flares” of AD, sleepless nights, poor school and work performance, absenteeism from personal and social activities, and psychological and social impact can adversely affect health in general [14]. Co-morbidities and secondary complications such as bacterial and viral skin infections, obesity, and hospitalisations increase burden [24]. Self and carer distress and exhaustion lead to under-treatment, AD exacerbation, and increased helplessness [59]. School-aged children with AD report increased bullying, teasing, and social isolation [58]. Dermatological conditions impact self-esteem throughout childhood which persists into adulthood [58,59]. Accessible, culturally appropriate mental health care and social support must remain a priority to optimise health outcomes. This is particularly important in certain subpopulations, including First Nations Peoples and refugees, who carry disproportionate mental health burdens and face enduring barriers to receiving care [60].

## 8. Conclusions

This narrative review of the burden of disease and unmet needs in the diagnosis and management of AD in diverse Australian ethnicities highlights gaps both in the current literature and in Australian healthcare; it aims to provide direction and suggestions for future research. AD is a heterogeneous disease that shows great variability among different ethnic and racial groups. It requires better clinical recognition across ethnicities and more personalised management approaches. There is likely under-recognition and under-treatment of AD amongst First Nations Peoples reflecting and in turn contributing to healthcare disparities that exist in the Australian health system. Children of immigrant Asian parents born in Australia are disproportionately affected by atopic disease suggesting a gene-environment factor is at play and this is also an area that warrants further exploration in order to develop models for prevention.

Multilevel health services have a responsibility to address the burden of disease and barriers to treatment of AD in order to provide culturally appropriate and accessible services that serve all people with skin of colour in Australia. This is needed to provide early, timely, and effective treatment of AD, its co-morbidities, and respective psychosocial needs. An increasing body of literature describes underreporting of race and ethnicity, and the overrepresentation of White individuals in prevalence studies, basic science research, and clinical trials. Further research to look for specific molecular targets in skin of colour is important to assist with personalised treatment for multi-ethnic populations. Improved understanding of the unique clinical phenotype of AD in different ethnic and racial groups, the intricate genetic and environmental interactions that influence susceptibility to AD and other atopic diseases, as well as the response of AD to current therapies in each particular subpopulation is, therefore, crucial to ensure appropriate management of an increasingly diverse Australian patient population.

## Figures and Tables

**Table 1:** Prevalence and incidence estimates of AD in the Australian Aboriginal and Torres Strait Islander peoples from the literature.

Study	Sampling Frame	Sample Size and Characteristics	Measurement and Definition Used	Prevalence/Incidence Estimates	Comparative Data with Other Racial Populations
Haggett, M.G., et al. (2021), [22]	Patient encounters with the primary Country Health Service visiting dermatology specialist in the Kimberley region between January 2012 and January 2017	2281 ‘episodes of care’ for a total of 1459 unique patients Median age yrs (Inter Quartile Range (IQR)) Total: 51 (35, 62) Aboriginal: 39 (19.8, 54.3)	Dermatologist diagnosis	Prevalence of Aboriginals N = 80/430 (19%) Number of encounters (% of all encounters)	Prevalence of Non-aboriginals N = 310/1851 (17%) Number of encounters (% of all encounters)
Blake, T.L., et al. (2020), [25]	Indigenous respiratory reference value (IRRV) study from eight communities (Queensland n = 7, Northern Territory (NT) n = 1) between June 2015 and November 2017. The study was a multicentre, cross-sectional cohort of Indigenous children and young adults (aged 3–25 years old)	889 Indigenous participants Median age yrs (IQR) 10.3 (7.2–13.3)	The International Study of Asthma and Allergies in Childhood (ISAAC) questionnaire Medical records information	Prevalence: ISAAC: 51/881 (5.8%) Medical records: 55/881 (6.2%) Both: 18/881 (2.0%)	None
Collaro, A.J., et al. [26]	Cross-sectional data of First Nations Australian children and young adults aged 5–25 years from the IRRV study	909 Indigenous participants Median age yrs (IQR) 11.0 (8.2, 13.8)	ISAAC questionnaire	Prevalence: ISAAC: 48/909 (5%)	None
Glasgow, N., et al. [27]	New entrants to kindergarten classes in all primary schools in the ACT in 1999, 2000 and 2001	Indigenous n = 217 Non-indigenous n = 10,604 Median age of 8 years, range 4−13 years	Ever eczema Questionnaire Has your child ever had eczema?	Indigenous Ever prevalence 50/202 (25%)	Non-indigenous Ever prevalence 3273/10,181 (32%)
Willems, A., et al. [28]	The Registrar Clinical Encounters in Training (ReCEnT) project records Australian GP registrar’s clinical and educational experience over these training terms.	2010–2019, 2783 registrars (96.1% response rate) provided data from 381,180 consultations, within which 595,412 problems were managed from all ages.	Problem/diagnosis of AD. Problems/diagnoses coded as ‘dermatitis, atopic’, ‘eczema’, and ‘eczema, infantile’	Eczema 39/10,055 problems (0.4%)	Eczema 3036/540,148 problems (0.6%)
Weber, H.C., et al. [29]	Children in North West Tasmania, students in grades 1 and 2 (average ages 6–8 years) were recruited from participating schools	1075 children 115 Indigenous 918 Non-indigenous	ISAAC questionnaire	Indigenous Prevalence 51/115 (44.4%)	Non-indigenous Prevalence 321/918 (35%)
Heyes, C., et al. [30]	Demographic data from each patient using public, outpatient services at each of the four hospitals in Perth between 1 of January 2010 and 31 July 2010 is retained on South Metropolitan Area Health Service and North Metropolitan Area Health Service databases	9639 ‘episodes of care’ for a total of 4873 patients over the 7-month period from 1 January 2010 to 31 July 2010. Median age yrs (IQR) Indigenous patients 22 (7, 43) Total patients 48 (24, 65)	Dermatology diagnosis	13/94 (14%)	None
Footprints in Time—The Longitudinal Study of Indigenous Children (LSIC) Key Summary Report from Wave 1—2009 [31]	The reference population was Aboriginal and Torres Strait Islander children living in Australia and born between December 2003 and November 2004 (Child cohort), and between December 2006 and November 2007 (Baby cohort).	1687 parents or primary carers of an Indigenous child.	Parent report	Prevalence of around 11% in both cohorts (baby and child)	None

## Data Availability

Data is contained within the article.
